# Impact of COVID-19 on Mental Health in Nursing Students and Non-Nursing Students: A Cross-Sectional Study

**DOI:** 10.3390/nursrep15080286

**Published:** 2025-08-06

**Authors:** Verena Dresen, Liliane Sigmund, Siegmund Staggl, Bernhard Holzner, Gerhard Rumpold, Laura R. Fischer-Jbali, Markus Canazei, Elisabeth Weiss

**Affiliations:** 1 Department of Psychology, University of Innsbruck, 6020 Innsbruck, Austrialaura.fischer-jbali@uibk.ac.at (L.R.F.-J.); markus.canazei@uibk.ac.at (M.C.); elisabeth.weiss@uibk.ac.at (E.W.); 2 Department of General and Social Psychiatry, University Hospital for Psychiatry I Medical University of Innsbruck, 6020 Innsbruck, Austria; bernhard.holzner@tirol-kliniken.at; 3 Department of General and Social Psychiatry, University Hospital for Psychiatry II Medical University of Innsbruck, 6020 Innsbruck, Austria; gerhard.rumpold@tirol-kliniken.at

**Keywords:** nursing students, non-nursing students in other academic majors, anxiety, depression, stress, mental stress and strain in internships

## Abstract

**Background/Objective**: Nursing and non-nursing students experience high stress levels, making them susceptible to mental health issues. This study compared stress, anxiety, and depression between these two groups after 2 years of the COVID-19 pandemic. Additionally, it explored the relationship between perceived helplessness, self-efficacy, and symptoms of mental stress and strain resulting from challenging internship conditions for nursing students. **Methods**: This cross-sectional study included 154 nursing students (mean age = 22.43 years) and 291 non-nursing students (mean age = 27.7 years). Data were collected using the Depression Anxiety Stress Scales (DASS-21), Perceived Stress Scale-10 (PSS-10), and a questionnaire on mental stress and strain. **Results**: Nursing students reported significantly higher scores in the DASS-21 subscales depression (ηp^2^ = 0.016) and anxiety (ηp^2^ = 0.037), and global stress (PSS-10; ηp^2^ = 0.029) compared to non-nursing students, but no significant difference on the DASS-21 Stress subscale. The observed group differences in the present study may be partially attributed to group differences in demographic factors. Helplessness correlated strongly with nearly all scales of mental stress and strain during internships (all *p*’s < 0.001), while self-efficacy showed a strong negative correlation with non-occupational difficulties, health impairment, and emotional problems (all *p*’s < 0.001). **Conclusions**: Nursing students experience elevated depression, anxiety, and perceived stress levels compared to non-nursing students. Stronger feelings of helplessness and lower confidence in their ability to overcome challenges were strongly correlated with mental stress and strain during clinical training. Targeted interventions such as cognitive behavioral training and stress management should be integrated into nursing curricula to enhance resilience and coping strategies.

## 1. Introduction

Starting a course of study, whether at university or in the care sector, is a challenging phase of life for young adults. It is associated with various stressors, including moving away from home, facing more demanding learning content and lectures, and making new social contacts. Psychological stress, time pressure, and a heavy workload are also common stressors for university students [1,2]. For nursing students, these challenges are compounded by additional unique stressors experienced during clinical training [3,4]. While clinical internships provide valuable insight into the nursing profession, they can also present emotional challenges, such as witnessing patient suffering, conflicts with family members, and patient deaths. These experiences often result in emotional and physical stress and fatigue [5,6]. A survey conducted in Austria found that 39% of nursing students reported sometimes experiencing physical and/or psychological stress during their internship, with 38% reporting experiencing this stress often or always [7]. This highlights the mental stress and strain inherent in both the nursing profession and its training.

The COVID-19 pandemic introduced further stressors for students such as the abrupt transition from face-to-face learning to distance learning, virtual exams, suspended internships, and closed university buildings. According to recent research, these changes led to increased uncertainty, an overwhelming flow of information through online/distance learning platforms, and a greater sense of loneliness among students, contributing to heightened levels of stress, anxiety, and depression among students [8,9,10,11,12,13,14]. However, nursing students in clinical training faced additional pandemic-related challenges, including increased work demands, intensive working hours, limited staffing, equipment shortages, rapid changes in hospital protocols, and inadequate information. They also experienced anxiety about their own health and the risk of spreading the disease to family and others [15,16].

Previous research has shown that both nursing students and non-nursing students in other academic majors experienced elevated stress levels during the COVID-19 pandemic. For instance, meta-analyses by Wang et al. [14] and Fang et al. [9] found prevalence rates of 23% and 31%, respectively, for stress symptoms among non-nursing students during the pandemic. In contrast, the meta-analysis by Zheng, Jiao, and Hao [17] estimated the stress prevalence rate among nursing students to be around 62%. These findings suggest that nursing students may be disproportionately affected by stress compared to their peers in other academic disciplines.

Stress is a known predictor of mental disorders and can also have both short- and long-term consequences for physical and mental health [18,19]. Previous meta-analyses have consistently reported higher prevalence rates of depressive and anxiety symptoms among nursing students compared to the general student population. For example, Mulyadi et al. [10] found that approximately 52% of nursing students reported depressive symptoms during the COVID-19 pandemic, while Wang et al. [14] and Fang et al. [9] reported prevalence rates of 37% and 32% respectively, among non-nursing students. Similarly, Vo et al. [2] found that about 60% of nursing students experienced anxiety levels ranging from mild to extreme, compared to 28–29% of non-nursing students [9,14]. Despite these findings, few studies have directly compared nursing students to the general student body in terms of stress levels and mental health outcomes. For example, Bartlett, Taylor, and Nelson [20] found that nursing students reported statistically significant higher levels of stress, anxiety, sleep disturbance, and stress-related illness than the general student population. In contrast, Black Thomas [21] observed that during the COVID-19 pandemic in 2021, nursing students reported statistically significant higher stress levels but lower depressive symptoms than their non-nursing peers in other disciplines. Based on qualitative interviews, the author hypothesized that a multifactorial combination of social support, resilience, and post-traumatic growth may explain this discrepancy.

One concept that may help explain these differences is “learned helplessness”, a psychological state characterized by a perceived lack of control over adverse situations, which can lead to passivity, reduced motivation, and depressive symptoms [22,23]. Learned helplessness is particularly relevant in the context of nursing education, where students often face high-stress environments, unpredictable clinical situations, and limited autonomy during training. Understanding the role of learned helplessness in nursing students’ stress and mental health outcomes is critical, as it may provide insights into why they experience higher stress levels yet exhibit varying patterns of depressive symptoms compared to non-nursing students.

Despite the growing body of literature on the mental health challenges faced by nursing students, there remains a lack of direct comparisons between nursing students and non-nursing students in terms of stress, anxiety, and depression, particularly in the context of the COVID-19 pandemic. Furthermore, few studies have explored the psychological mechanisms, such as learned helplessness and self-efficacy, that may underlie these differences. This study seeks to address this gap by examining the relationship between perceived helplessness, self-efficacy, and psychological symptoms in nursing students, with a focus on the unique stressors they face during clinical training. By doing so, this study aims to contribute to the understanding of mental health disparities between nursing and non-nursing students and to identify potential areas for intervention to support the well-being of nursing students. Specifically, the current study aims to compare the stress, anxiety, and depression scores of nursing students with those of the general student population after 2 years of the COVID-19 pandemic (from March 2022 to May 2023). Our hypothesis is that nursing students will have statistically significant higher levels of stress, depression, and anxiety than non-nursing students in other academic majors. In addition, we will examine the differences in perceived helplessness and self-efficacy between the two groups. Furthermore, the study will examine the relationship between perceived helplessness and self-efficacy and psychological symptoms of mental stress and strain due to difficult and unhealthy working conditions in nursing students’ internships. Finally, the relationship between demographic factors, working hours, and stress-related outcomes in nursing students will be explored.

## 2. Materials and Methods

This study utilizes cross-sectional data for non-nursing students from the baseline survey of Care4Stress Psychoeducation study [24]. The Care4Stress project aimed to investigate the acceptability and efficacy of a 7-week app-based passive psychoeducation stress-management program to promote adaptive emotion regulation and coping skills in nursing students and non-nursing students (for non-nursing students please see [24]). Participation in the study was voluntary and informed consent was obtained from all students prior to participation. The study was in accordance with the 1964 Declaration of Helsinki and was approved by the local ethics committee. The sample comprised German-speaking non-nursing university students from various faculties in Austria and Germany. They were recruited via posts in Facebook groups and official students mailing list of the University of Innsbruck (for all study programs). Bachelor students of psychology received course credits for participation in this study. The nursing students were invited via a letter forwarded by the head of their respective Austrian and German nursing school. Data collection for the non-nursing sample was conducted between May 2022 and July 2022 and between October 2022 and December 2022, while data for the nursing sample were collected from March 2022 to May 2023. A total of 445 students participated in the survey, including 154 nursing students (116 female, 38 male) and 291 non-nursing students (223 female, 65 male, and 3 gender diverse) from other disciplines (i.e., 75% were studying psychology). Of the general non-nursing students, 85% (*n* = 247) were undergraduate students, while 13% (*n* = 39) were enrolled in a master program and 2% (*n* = 5) in a PhD program. Most of the nursing students worked more than 30 h per week during their internship.

Psychometric assessment was conducted using an online survey administered through LimeSurvey [25] for non-nursing students and through CHES (Computer-based Health Evaluation System; [26]) for nursing students and included information on sociodemographic data, stress, anxiety and depression scores. In addition, two questionnaires were used for nursing students to assess mental stress and strain during their internships.

### 2.1. Study Materials

#### 2.1.1. Depression Anxiety Stress Scales (DASS-21; [27]): German Version [28,29]

The participant’s mental health was evaluated using the self-report screening instrument DASS-21. The questionnaire comprises three subscales, each with seven items, that assess depression, anxiety, and stress. Participants rated how they felt in the past week on a four-point Likert scale ranging from 0 (never) to 3 (most of the time). Total scores for each scale are computed by summing up all items of a scale.

#### 2.1.2. Perceived Stress Scale-10 (PSS-10; [30]): German Adaption [31]

The PSS-10 is used to assess an individual’s perception of stress. It measures the degree to which situations in the person’s life over the past month are perceived as stressful, unpredictable, and uncontrollable. The PSS-10 comprises ten items, divided into two subscales, namely, perceived helplessness (6 items) and perceived self-efficacy (4 items). The Helplessness subscale reflects individual’s perception of stress due to feelings of a lack of control, e.g., “In the last month, how often have you felt nervous and stressed”. The Self-efficacy scale measures an individual’s perception of confidence to handle problems, e.g., “In the last month, how often have you felt that things were going your way”. Items are answered on a 5-point frequency rating scale ranging from 0 (never) to 4 (very often). Outcome measures of the two subscales were derived from summing up the items. For the calculation of the Global score, the four items of the Self-efficacy subscale were inverted and added with the rating of the Helplessness subscale, with higher scores reflecting greater levels of perceived stress.

#### 2.1.3. Questionnaire on Mental Stress and Strain [32]

A questionnaire from the German Social Accident Insurance Institution for the Health and Welfare Services (BGW) was used to assess the mental stress and strain of nursing students during their internship. The questionnaire was developed specifically for nurses. The first part of the mental stress questionnaire aims to identify difficult and unhealthy working conditions in inpatient care. The participants rate 22 statements on a 5-point Likert scale ranging from 1 (no, not at all) to 5 (yes, exactly). The following 5 scales can be calculated: Quantitative workload (5 items), Qualitative workload (5 items), Work organization (3 items), Social work climate (6 items), and Non-work situation (3 items). The scale means were used in the statistical analysis. The second part of the questionnaire on mental strain assesses whether symptoms typical of stress are statistically significant pronounced in nursing staff. Emotional symptoms are assessed on the basis of 10 items including work motivation (1 item), job dissatisfaction (2 items), reactive shielding (2 items), aversion to residents (2 items), and emotional exhaustion (3 items). The rating is based on a 7-point Likert scale (1 = “strongly disagree” to 7 = “strongly agree”). Additionally, health impairment and symptoms of fatigue were measured using 5 questions on a 5-point Likert scale (1 = almost every day to 5 = never). Again, the scale means were used in the statistical analysis. The two questions related to non-work situation were excluded from this questionnaire as they were identical to the questions in the mental stress questionnaire.

### 2.2. Statistics

All statistical procedures were performed in SPSS 26. As the normal distribution assumption was violated, group differences in age were calculated using the Mann–Whitney U test. Other demographic characteristics of the two groups were compared by means of Pearson’s chi-square tests. Differences between non-nursing students and nursing students in the in the Depression-Anxiety-Stress Scales (DASS-21), Perceived Stress Scale-10 (PSS-10) were calculated using univariate and multivariate analysis of variance (M)ANOVAs. Effect sizes in (M)ANOVAs are reported as partial eta-squared (ηp^2^), with ηp^2^ ranging from 0.01 to 0.06 representing a small effect, from 0.06 to 0.14 a medium effect, and at least 0.14 a large effect. Pearson’s correlations were used to explore the relationship between the scales of the mental stress and strain questionnaire and the PSS-10 subscales Helplessness and Self-efficacy in nursing students. The relationship between the scales of the DASS-21, PSS-10 and the mental stress and strain questionnaire and demographic variables in nursing students were assessed with Spearman correlations. For the statistical significance test, the alpha level was set a priori at 0.05.

## 3. Results

Sociodemographic and study-related information for the non-nursing and nursing student groups is shown in Table 1.

Nursing students were statistically significant older than non-nursing students in other academic majors (U(n1 = 291, n2 = 154) = 18.596, z = −2.87, *p* = 0.004). Additionally, nursing students were more likely to be in a partnership (χ*^2^*(1, N = 445) = 10.76, *p* = 0.001, φ = 0.16), have children (χ*^2^*(2, N = 445) = 78.75, *p* < 0.001, φ = −0.42), and smoke (χ*^2^*(1, N = 445) = 44.67, *p* < 0.001, φ = −0.32) compared to the non-nursing group. In addition, nursing students reported a statistically significant higher frequency of physical illness (χ*^2^*(1, N = 445) = 20.26, *p* < 0.001, φ = −0.21) and psychological/psychotherapeutic treatment (χ*^2^*(2, N = 445) = 13.56, *p* = 0.001, φ = 0.18) compared to non-nursing students in other disciplines. However, there was no statistically significant difference between the groups in terms of regular alcohol consumption (χ*^2^*(1, N = 445) = 1.48, *p* = 0.224), frequency of mental illness (χ*^2^*(1, N = 445) = 2.334, *p* = 0.127), or gender distribution (χ*^2^*(2, N = 445) = 1.848, *p* = 0.397). Table 2 shows the mean values and standard deviations for the subscales of the DASS-21 (Depression, Anxiety, and Stress) and the PSS-10 scores (Global, Helplessness, Self-efficacy).

**Figure 1:**
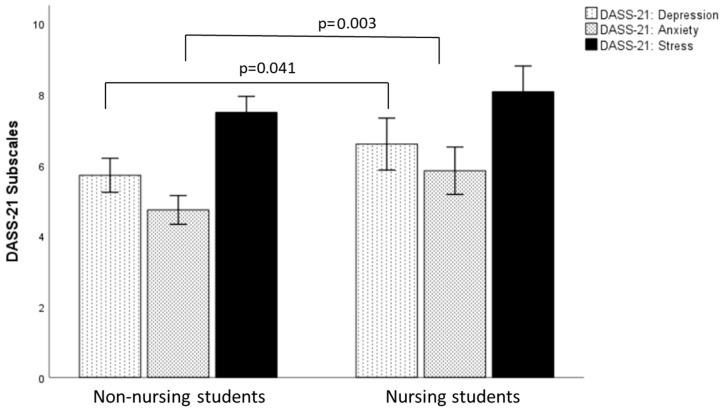
Group differences in the DASS-21 subscales. Note: M = Mean, 95% Confidence intervals, DASS-21 = Depression-Anxiety-Stress-Scale.

A multivariate analysis of covariance (MANCOVA) carried out with the three DASS-21-scales with group (nursing students, non-nursing students) as between-subjects factor and age as the covariate was statistically significant (*F*(3, 440) = 6.156, *p* < 0.001, ηp*^2^* = 0.040), see Figure 1 for details. Follow-up univariate one-way ANCOVAs were performed. A Bonferroni adjustment was made such that significance was accepted at *p* < 0.0167. Nursing students had statistically significant higher scores in adjusted means for the Depression scale (*F*(1, 442) = 7.157, *p* = 0.008, ηp*^2^* = 0.016) and the Anxiety scale (*F*(1, 442) = 17.131, *p* < 0.001, ηp*^2^* = 0.037) than non-nursing students in other disciplines. No statistically significant difference could be found between the two groups in the adjusted means for the DASS-21 Stress scale (*F*(1, 442) = 3.630, *p* = 0.057, ηp*^2^* = 0.008).

An ANCOVA using the PSS-10 global score and age as the covariate showed a statistically significant higher Global stress score in nursing students than non-nursing students (*F*(1, 442) =13.348, *p* < 0.001, ηp^2^ = 0.029).

**Figure 2:**
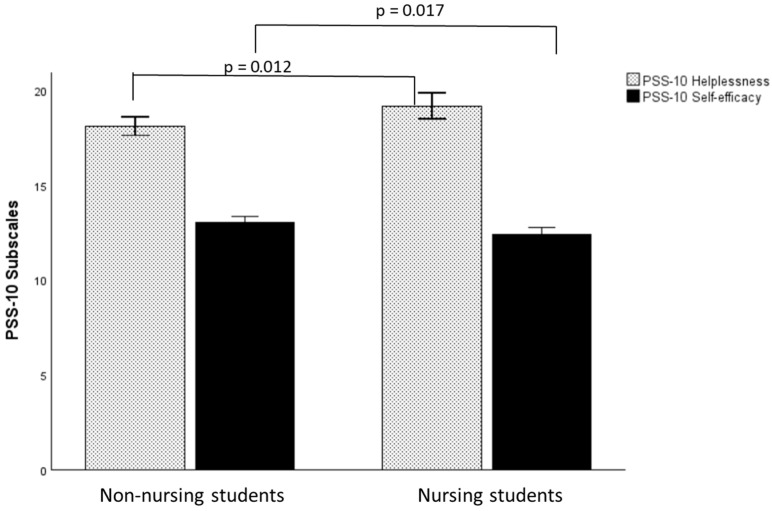
Group differences in the PSS-10 subscales. Note: *M* = Mean, 95% Confidence intervals, PSS-10 = Perceived Stress Scale-10.

A MANCOVA carried out on the two subscales of the PSS-10 with group as between-subjects factor and age as the covariate was statistically significant (*F*(2, 441) = 7.191, *p* < 0.001, ηp^2^ = 0.032). Follow-up univariate one-way ANCOVAs were performed. A Bonferroni adjustment was made such that significance was accepted at *p* < 0.025. Nursing students had statistically significant higher scores in the Helplessness scale (*F*(1, 442) = 9.349, *p* = 0.002, ηp*^2^* = 0.021) and lower scores in the Self-efficacy scale (*F*(1, 442) = 12.917, *p* < 0.001, ηp*^2^* = 0.028) than non-nursing students in other academic majors, see Figure 2.

During internships, nursing students experienced high levels of mental stress and strain measured with the questionnaire for mental stress and strain. Subscales for mental stress showed the following: Quantitative workload (M = 3.6, SD = 0.77), Qualitative workload (M = 2.46, SD = 0.72), Work organization (M = 3.18, SD = 0.91), Social work climate (M = 2.73, SD = 0.83), Non-work situation (M = 2.87, SD = 0.83). Subscales for mental strain showed the following: Emotional symptoms (M = 3.54, SD = 1.17), Health impairment (M = 3.52, SD = 0.91). Significant positive correlations between the PSS-10 subscale Helplessness and all scales of the mental stress and strain questionnaire were shown (all *p*’s < 0.003) except for the Work organization scale. Additionally, Self-efficacy was negatively correlated with Quantitative workload (*p* = 0.04), difficulties in Non-work situations (*p* < 0.001), Health impairments/fatigue symptoms (*p* < 0.001), and Emotional problems (*p* < 0.001). However, no statistically significant relationships were found between Self-efficacy and Qualitative workload, Work organization, and Social work climate in nursing students. Table 3 shows the correlation between the scales of the mental stress and strain questionnaire with the PSS-10 subscales Helplessness and Self-efficacy in nursing students.

Examining the relationship between demographic factors, working hours, and stress-related outcomes in nursing students, we observed several noteworthy findings. A small but significant negative correlation was identified between age and the DASS-21 Depression score (*r* = −0.213, *p* = 0.008) as well as the DASS-21 Anxiety score (*r* = −0.254, *p* = 0.002), suggesting that older participants tend to report slightly lower levels of depressive and anxiety symptoms. Additionally, age was negatively correlated with the PSS-10 Global score (*r* = −0.194, *p* = 0.023) and positively correlated with the PSS-10 Self-efficacy subscale (*r* = 0.202, *p* < 0.05), indicating that older participants reported slightly lower overall stress levels and perceived themselves as slightly more self-efficacious. Furthermore, a small positive correlation was found between age and Social work climate (*r* = 0.165, *p* = 0.42).

No significant correlations were observed between the number of children and any of the questionnaire scores.

Regarding working hours, nursing students who worked more hours demonstrated slightly higher scores on the DASS-21 Depression scale (*r* = 0.194, *p* = 0.016), the DASS-21 Stress scale (*r* = 0.201, *p* = 0.012) and the PSS-10 Global score (*r* = 0.244, *p* = 0.002). Additionally, working hours were positively correlated with the PSS-10 Helplessness scale and negatively correlated with the PSS-10 Self-efficacy scale (*r* = −0.193, *p* = 0.017). These results indicate that longer working hours are associated with higher levels of stress and depression as well as a greater sense of helplessness and less self-efficacy.

Finally, with respect to the mental stress and strain scale, nursing students working more hours exhibited slightly higher scores on the scales for mental stress in Non-work situation (*r* = 0.168, *p* = 0.037) and health impairments (*r* = 0.205, *p* = 0.011). Table 4 shows the correlation between the scales of the DASS-21, PSS-10, and the mental stress and strain questionnaire and demographic variables in nursing students.

## 4. Discussion

In the present study, nursing students reported significantly higher depression and anxiety scores on the DASS-21 compared to non-nursing students in other disciplines, although the effect size of this difference was small. Despite the fact that most previous studies reported higher anxiety levels in nursing students than in non-nursing students [9,10,20], the results regarding depression were much more heterogeneous. For example, the meta-analysis by Tung et al. [33] and the study by Bartlett, Taylor, and Nelson [20] found a statistically significant lower prevalence of depressive symptoms in nursing students than Mulyadi et al. [10] or Fang et al. [9]. One possible contributing factor to these differences is the impact of the COVID-19 pandemic, which has been associated with increased workload in the nursing profession and heightened stress levels, potentially affecting mental health outcome [34,35,36]. For example, a meta-analysis of 23 studies found that more than 20% of nurses experienced symptoms of depression and over 33% reported anxiety symptoms during the pandemic [36]. Nursing students are exposed to specific stressors, particularly during clinical training [3,4]. Commonly reported stressors include time pressure [37,38,39], lack of recognition [40,41], physical stress (e.g., [42]), and emotional and psychological stress [43,44], particularly in the field of geriatric nursing, when dealing with chronic illnesses such as dementia [45] and death [46]. Consistent with the literature [2,9,14,20,29], nursing students in the present study also reported statistically significant higher scores on the PSS-10 Helplessness subscale and lower scores on the Self-efficacy subscale than non-nursing students, although the effect size of this difference was small, while there were no statistically significant differences between the two groups on the Stress scale of the DASS-21. One possible explanation for the lack of significant differences in the DASS-21 Stress score between nursing and non-nursing students lies in different stress conceptualizations of the two instruments. The DASS-21 Stress subscale focuses on acute emotional and physiological symptoms of stress (e.g., nervousness, restlessness) over a short time frame (past week), whereas the PSS-10, which revealed significant differences in the present study, assesses helplessness and self-efficacy as two additional dimensions of stress, over a longer period (past month) [28,31]. This difference in scope and focus of the two scales may explain the discrepancy in the findings. Furthermore, the DASS-21 may be less sensitive to the chronic, cumulative stressors of nursing students, such as academic pressure and clinical responsibilities, which are better captured by the PSS-10. Additionally, nursing students may develop coping strategies that mitigate acute stress symptoms, resulting in comparable DASS-21 scores despite differing underlying stressors. The PSS-10’s ability to capture the psychological burden of helplessness and its broader time frame may make it more suitable for assessing the unique stress profiles of nursing students. These findings align with prior research showing that the PSS-10 is more sensitive to the stress experiences of nursing students compared to the DASS-21 [2]. Cultural and contextual factors, such as the development of professional identity and ethical challenges, may also shape stress responses in ways not fully grasped by the DASS-21. Future research should explore alternative measures or qualitative approaches to better capture the nuanced stress experiences of nursing students.

The current study also identified a strong correlation between Helplessness and all scales measuring mental stress and strain during internships, except for the Work organization scale. Additionally, Self-efficacy was negatively correlated with Quantitative workload, difficulties in Non-work situations, Health impairments/fatigue symptoms and Emotional problems. However, no statistically significant relationships were found between Self-efficacy and Qualitative workload, Work organization, and Social work climate. Gibbons [47] showed that self-efficacy, dispositional control, and support were important predictors of well-being in nursing students, while avoidance coping had a negative impact on mental health. The observed group differences in the present study may be partially attributed to demographic factors. The higher average age of the nursing students compared to non-nursing students in other disciplines, along with additional responsibilities such as partnerships, children, and more frequent physical illnesses, may also explain the higher levels of anxiety, depression, and stress reported by nursing students. These findings align with previous research, which has documented that nursing students are often older, more likely to have families with children, and report more non-occupational stress and stress-related illnesses, such as migraine or upper respiratory tract infections [20,21,48,49,50].

Furthermore, our results suggest that older nursing students tend to report lower levels of depressive and anxiety symptoms, as well as lower overall stress levels, while perceiving themselves as more self-efficacious. These observations are consistent with prior studies that propose older nursing students may benefit from more developed coping mechanisms and greater life experience, which could enable them to manage stress and mental health challenges more effectively [51]. These advantages may stem from their ability to balance multiple roles and responsibilities. Additionally, older students may cultivate more supportive relationships in their work environments, which could enhance their resilience. However, this interpretation contrasts with earlier research suggesting that having children could be a significant source of responsibility and stress [52]. Notably, in our study, no significant relationship was found between the number of children and stress-related outcomes.

Our findings also highlight an association between longer working hours during clinical training and elevated levels of stress and depression. Nursing students who worked more hours reported feeling less capable of managing stress and experiencing a greater sense of helplessness. These results are consistent with prior studies in nurses and nursing students [53,54], particularly those conducted during the COVID-19 pandemic, which documented additional stressors faced by healthcare workers [55,56]. These stressors included increased work demands such as intense schedules, limited staffing, lack of personal protection equipment (PPE) at work, rapidly changing hospital protocols, and insufficient information. Such conditions were linked to heightened psychological distress and insomnia especially among female physicians, nurses/nursing students, and other frontline health care professionals [15,16,57]. Furthermore, previous research has highlighted the significant impact of the rapidly evolving clinical landscape and disruptions to nursing education during the pandemic. These disruptions, including the shift to distance learning and reduced opportunities for hands-on clinical practice, have been associated with heightened stress, anxiety, and feelings of unpreparedness among nursing students [58,59,60]. Unfortunately, our study does not include data on whether the nursing students missed practical training during earlier waves of the COVID-19 pandemic. Such interruptions could have introduced additional sources of anxiety and insecurity, particularly as students transitioned back to clinical settings during the pandemic [58,59,60].

Interestingly, pre-pandemic research noted that nursing students and the general student population did not differ significantly in terms of employment status, with nursing students often working fewer hours than their peers [20]. Additionally, non-nursing students were more likely to participate in intensive leisure activities such as sports [20], which has been identified as a significant stressor due to time demands and role conflicts [61]. However, it is important to note that the current study was conducted during the COVID-19 pandemic, a period when many non-nursing students had less possibilities to participate in intensive leisure activities and were not able to pursue a part-time job due to the restrictions during the COVID-19 pandemic. This context limits the ability to directly compare the influence of working hours between nursing and non-nursing students.

Finally, it is important to consider that other factors unique to the nursing profession may be associated with the observed differences in stress-related outcomes between nursing students and their non-nursing peers. These factors include concerns about future employment, role ambiguity, attitudes of medical personnel and patients, difficulties in bridging theoretical knowledge with practical application, and anxieties related to the clinical environment [62]. Such stressors may intensify the challenges faced by nursing students, further distinguishing their experiences from those of the general student population.

Against this background, it becomes even more crucial to offer preventive measures and psychological support services that are specifically tailored to nursing students. Internet-based training programs grounded in evidence-based approaches, such as cognitive behavioral therapy, show promise as accessible and cost-effective solutions for mitigating stress and improving mental health outcomes [63]. Furthermore, integrating skill development into nursing curricula, as emphasized by Frenzel, Götz, and Pekrun [64], could enhance subjective control beliefs and emotional regulation, preparing students to better manage both academic and workplace stress. Additionally, institutions should evaluate the effectiveness of hybrid or online learning models introduced during the pandemic and explore how these can be optimized to support both academic and emotional well-being. Future studies should rigorously evaluate the impact of these curricular changes, not only on students’ academic experiences but also their ability to manage workplace stress after graduation. Longitudinal studies are particularly needed to examine how these interventions influence resilience, job satisfaction, and career longevity in the nursing profession. By proactively addressing these challenges, nursing education can better prepare future health professionals to meet high working demands while maintaining their mental health and professional satisfaction.

### Limitations

The present study has several limitations that should be taken into account when interpreting the findings. First, the sample size of the nursing student group was relatively small, primarily due to recruitment challenges. Only a small proportion (2%) of the nursing schools contacted forwarded the study invitation to their students, which may have introduced sampling bias. As a result, the nursing students who participated in the study may not fully represent the broader nursing student population, potentially influencing the observed results. Furthermore, we were unable to evaluate the similarities or differences between nursing programs across the participating schools, which limits the extent to which the findings can be generalized to nursing students in other educational or cultural contexts.

Second, the group of non-nursing students consisted predominantly of psychology students, which may also affect the generalizability of our results. Psychology students may have greater awareness of mental health and stress-coping strategies compared to the general student population. This imbalance in academic disciplines may have introduced a bias in the comparison of stress levels and coping mechanisms between nursing and non-nursing students.

Third, the gender distribution in both samples was heavily skewed with the majority of the participants identifying as female. While this reflects the gender distribution commonly observed in nursing and psychology programs, it limits the applicability of our findings to male students or those of other gender identities.

Fourth, cultural factors were not explicitly examined in this study. The participants were drawn from a specific geographic and cultural context, which may limit the applicability of the findings to nursing students in other regions or countries. Cultural differences in stress perception, coping mechanisms, and mental health stigma may influence the results, and future research should explore these factors to enhance cross-cultural relevance of the findings.

Fifth, the correlational/cross-sectional design of this study precludes any conclusions about causality and direction of influence between variables. While associations between stress, anxiety, and depression were identified, it is not possible to determine whether stress contributes to mental health challenges or whether pre-existing mental health conditions are associated with higher stress levels. Longitudinal studies are needed to better understand the temporal relationships between these variables and provide a more comprehensive understanding of the factors influencing nursing students’ mental health.

Sixth, previous research has demonstrated that both the DASS-21 and PSS-10 are reliable and valid instruments for assessing mental health in nursing and non-nursing student populations (e.g., [65,66,67,68,69,70,71]). However, the findings of the present study, in which the DASS-21 Stress subscale did not show significant differences between groups, highlight the importance of selecting measurement tools that are appropriately tailored to the specific characteristics of the population under investigation. The PSS-10, with its focus on perceived helplessness and self-efficacy as well as its longer assessment time frame, may be more effective in capturing the chronic stressors prevalent among nursing students. Future research should further explore the dynamic interplay between acute and chronic stress, along with the role of coping mechanisms, in shaping stress outcomes within this population.

Finally, the use of different survey platforms may have introduced potential biases. Variations in platform design or user interfaces could have influenced how participants engaged with the survey and responded to the questions. These differences may have affected the comparability of responses and the generalizability of the findings.

Future longitudinal studies, in particular, are recommended to address this limitation by tracking participants over time to observe changes in stress, anxiety, and depression. Such studies could incorporate repeated measures and control for potential confounding factors to clarify the temporal relationship underlying the observed associations.

## 5. Conclusions

The findings of our study contribute to the growing body of evidence suggesting that nursing school is a particularly stressful experience, especially during clinical training. Nursing students in our study reported elevated levels of depression and anxiety, as well as higher perceived stress compared to students in other disciplines. They also expressed stronger feelings of helplessness and lower confidence in their ability to overcome challenges, underscoring the need for targeted interventions to address the unique stressors faced by this population. While these findings highlight critical areas for action, they should be interpreted with caution due to the study’s limitations, such as the limited sample size, the prevalence of potential confounding factors, and the cross-sectional study design.

The long-term impact of the COVID-19 pandemic on the training of health professionals, including nursing students, must also be considered. The pandemic has underscored the importance of resilience training, crisis management skills, and adaptability in healthcare settings. Future nursing curricula should incorporate modules that address these competencies, preparing students to navigate high-stress environments and public health emergencies.

## Figures and Tables

**Table 1:** Sociodemographic and study-related information.

		Non-Nursing Students (*n* = 291)	Nursing Students (*n* = 154)	Total (*n* = 445)
Age (Years)	Minimum	18	16	16
	Maximum	46	62	62
	Mean (*SD*)	22.43 (3.61)	27.7 (10.99)	24.25 (7.5)
		*n* (%)	*n* (%)	*n* (%)
Gender	Female	223 (77)	116 (75)	339 (76)
	Male	65 (22)	38 (25)	103 (23)
	Gender diverse	3 (1)	0	3 (1)
Marital status	Single/divorced/ widowed	200 (69)	81 (53)	281 (63)
	Married/with a life partner	91 (31)	73 (47)	164 (37)
Level of education	Bachelor	247 (85)	-	-
	Master	39 (13)	-	-
	PhD	5 (2)	-	-
University courses	Psychology	217 (75)	-	-
	Other courses	74 (25)	-	-
Working-hours/week	<10 h	-	4 (3)	-
	10–19 h	-	5 (3)	-
	20–29 h	-	10 (6)	-
	>30 h	-	135 (88)	-
Children	Yes	3 (1)	43 (28)	46 (10)
	No	288 (99)	111 (72)	399 (90)
Number of children	No children 1–2 children ≥3 children	288 3 -	111 35 8	399 38 8
Smoking	Yes	33 (11)	59 (38)	92 (21)
	No	258 (89)	95 (62)	353 (79)
Alcohol consumption	Yes	57 (20)	23 (15)	80 (18)
	No	234 (80)	131 (85)	365 (82)
Current or past mental illness	Yes	34 (12)	26 (17)	60 (14)
	No	257 (88)	128 (83)	385 (86)
Current or past physical illness	Yes	26 (9)	38 (25)	64 (14)
	No	265 (91)	116 (75)	381 (86)
Psychological/psychotherapeutic/psychiatric treatment	Current	48 (17)	14 (9)	62 (14)
	Past	62 (21)	56 (36)	118 (27)
	No	181 (62)	84 (55)	265 (59)

Note: *SD* = Standard deviation, *n* = Number of participants.

**Table 2:** Mean values and standard deviations for the DASS-21 subscales and the PSS-10 subscales.

		Non-Nursing Students (*n* = 291)		Nursing Students (*n* = 154)		Total (*n* = 445)	
		*M*	*SD*	*M*	*SD*	*M*	*SD*
DASS-21	Depression	5.72	4.18	6.60	4.62	6.02	4.35
	Anxiety	4.74	3.53	5.84	4.21	5.12	3.81
	Stress	7.49	3.95	8.08	4.57	7.69	4.18
PSS-10 Global Score		29.10	6.47	30.82	5.86	29.69	6.32
PSS-10 Helplessness		18.12	4.32	19.20	4.29	18.50	4.34
PSS-10 Self-efficacy		13.08	2.76	12.38	2.59	12.81	2.72

Note: *M* = Mean, *SD* = Standard deviation, DASS-21 = Depression-Anxiety-Stress-Scale, PSS-10 = Perceived Stress Scale.

**Table 3:** Pearson’s correlation coefficients (*r*) between the scales of the mental stress and strain questionnaire and the PSS-10 subscales Helplessness and Self-efficacy in nursing students.

	Helplessness	Self-Efficacy
	**(*n* = 154)**	**(*n* = 154)**
**Mental Stress**		
Quantitative workload	0.272 **	−0.166 *
Qualitative workload	0.235 **	−0.084
Work organization	0.132	−0.079
Social work climate	0.226 **	−0.141
Non-work situation	0.329 **	−0.278 **
**Mental strain**		
Emotional symptoms	0.708 **	−0.457 **
Health impairment	0.550 **	−0.506 **

Note: Significance Level: * *p* < 0.05; ** *p* < 0.01.

**Table 4:** Spearman correlation coefficients (*r*) between the scales of the DASS-21, PSS-10, and the mental stress and strain questionnaire and demographic variables in nursing students.

	Age	Number of Children	Working Hours
**DASS-21**			
Depression	−0.213 **	0.129	0.194 *
Anxiety	−0.254 **	−0.099	0.148
Stress	−0.121	0.079	0.201 *
**PSS-10**			
Helplessness	−0.136	0.075	0.237 **
Self-efficacy	0.202 *	0.207	−0.193 *
Global score	−0.184 *	−0.057	0.244 **
**Mental Stress**			
Quantitative workload	0.013	−0.107	0.056
Qualitative workload	−0.152	−0.069	−0.007
Work organization	0.116	0.284	0.083
Social work climate	0.165 *	−0.008	−0.031
Non-work situation	0.090	−0.107	0.168 *
**Mental Strain**			
Emotional symptoms	−0.142	−0.139	0.145
Health impairment	−0.076	0.063	0.205 *

Note: Significance Level: * *p* < 0.05; ** *p* < 0.01.

## Data Availability

The datasets generated and/or analyzed during the current study are available from the corresponding author upon reasonable request.

## Public Involvement Statement

There was no public involvement in any aspect of this research.

## Guidelines and Standards Statement

This manuscript was drafted against the STROBE guidelines [72] for cross-sectional studies.

## Abbreviations

The following abbreviations are used in this manuscript:

COVID-19	Coronavirus disease of 2019
PhD	Doctor of Philosophy
Care4Stress	7-week app-based passive psychoeducation stress-management program
CHES	Computer-based Health Evaluation System
DASS-21	Depression Anxiety Stress Scales
PSS-10	Perceived Stress Scale-10
BGW	German Social Accident Insurance Institution for the Health and Welfare Services
SPSS 26	Statistical Package for the Social Sciences
(M)ANOVA	(Multivariate) Analysis of Variance
